# A Case of Retroperitoneal Synovial Sarcoma in Pregnancy Treated with Antepartum Doxorubicin plus Ifosfamide Chemotherapy

**DOI:** 10.1155/2021/9982171

**Published:** 2021-07-16

**Authors:** Bradley H. Sipe, Sarah G. Običan, Evita Henderson-Jackson, Nicole D. Riddle, Rikesh Makanji, Ricardo J. Gonzalez, Andrew S. Brohl

**Affiliations:** ^1^Department of Obstetrics and Gynecology, Morsani College of Medicine, University of South Florida, Tampa, Florida, USA; ^2^Department of Anatomic Pathology, H. Lee Moffitt Cancer Center and Research Institute, Tampa, Florida, USA; ^3^Department of Pathology, Morsani College of Medicine, University of South Florida, Tampa, Florida, USA; ^4^Ruffolo, Hooper, and AssociatesTampa, Florida, USA; ^5^Diagnostic Imaging, H. Lee Moffitt Cancer Center and Research Institute, Tampa, Florida, USA; ^6^Sarcoma Department, H. Lee Moffitt Cancer Center and Research Institute, Tampa, Florida, USA

## Abstract

We report a case of a 25-year-old pregnant woman diagnosed with a large, unresectable retroperitoneal synovial sarcoma. Successful neoadjuvant treatment with doxorubicin plus ifosfamide prepartum and continuing postpartum resulted in significant disease response allowing for later tumor resection. Following the first prepartum chemotherapy cycle, a decreased amniotic fluid index was noted, representing a potential complication of chemotherapy. Induction of labor was performed at 33 weeks gestation with excellent outcome in the newborn. This case highlights the complex medical decision-making process in the setting of cancer diagnosed during pregnancy, balancing oncologic and obstetric concerns, and to our knowledge is only the second reported case of synovial sarcoma treated with neoadjuvant cytotoxic chemotherapy in the antepartum period.

## 1. Case Presentation

A 25-year-old G3P0202 presented at 22w3d gestation for evaluation of a newly discovered retroperitoneal mass found during workup for abdominal pain. The patient had experienced one year of ongoing, worsening abdominal pain. She had a history of two prior preterm deliveries at 36 weeks gestation but otherwise was healthy with no chronic medical conditions.

**Figure 1:**
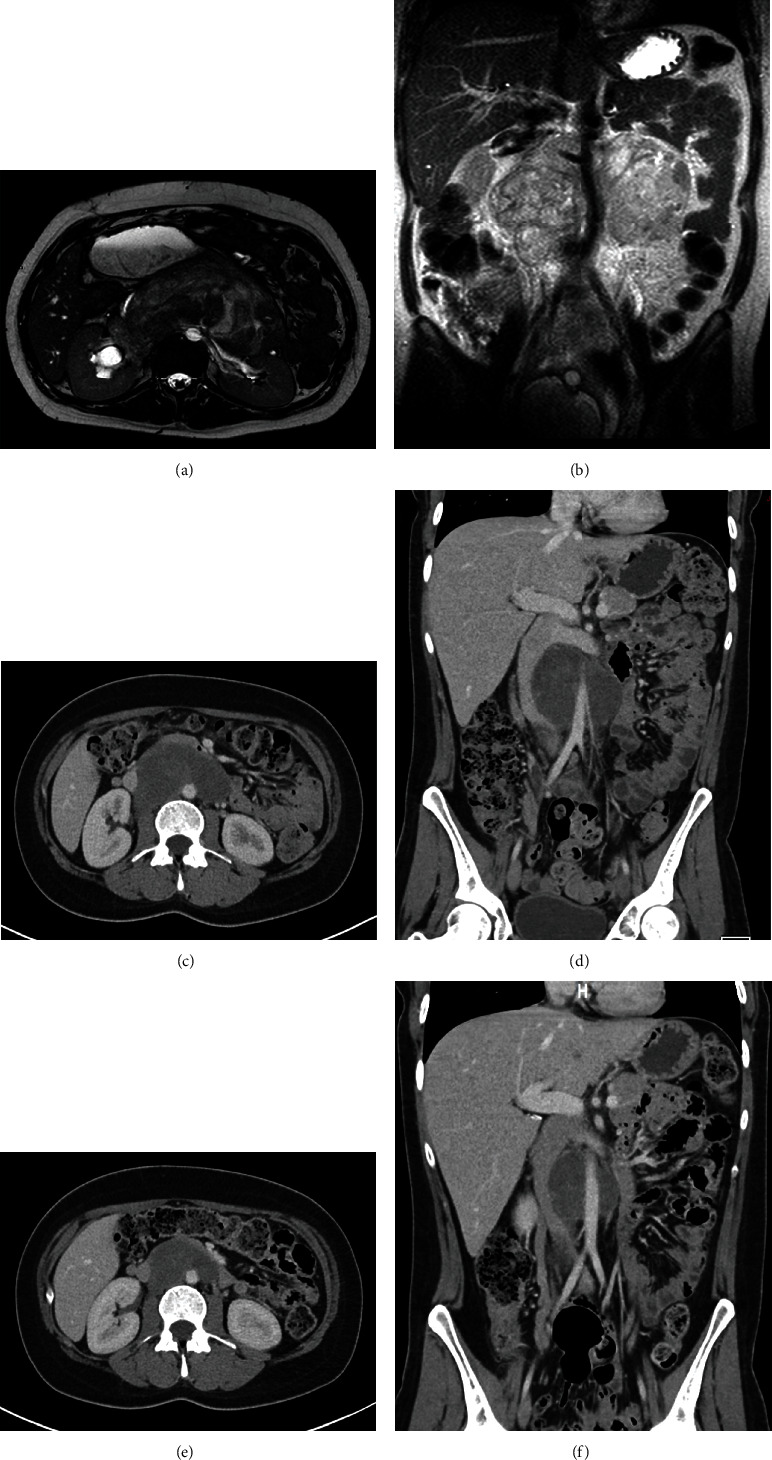
Radiology of synovial sarcoma case. Magnetic resonance imaging prior to treatment (a, b) demonstrating the large retroperitoneal mass. (a) Fluid sensitive axial image shows the mass partially encasing the abdominal aorta and compressing and displacing the inferior vena cava. Mild right hydronephrosis is evident. (b) T2 coronal image showing extensive encasement of the aorta and right renal artery by the large retroperitoneal mass. Computed tomography imaging post 3 cycles (c, d) and 6 cycles (e, f) of neoadjuvant chemotherapy demonstrating treatment response as evidenced by marked decrease in tumor size, decreased mass effect on the vena cava, resolution of right hydronephrosis, and deceased tumor density (suggestive of necrosis).

Magnetic resonance imaging (MRI) evaluation revealed a 13 cm transverse by 8 cm anterior-posterior by 9 cm craniocaudal smoothly marginated mass inferior to the pancreas which encased the left renal vessels, displaced the inferior vena cava (IVC) anteriorly, and partially encased the abdominal aorta (Figure 1). A computerized tomography (CT) guided biopsy was unable to be performed due to the surrounding anatomy and gravid uterus; therefore, a laparoscopic biopsy was planned. CT of the chest was performed showing no evidence of metastatic disease. The patient was readmitted for planned laparoscopic biopsy at 27w2d gestation. She was given corticosteroids for fetal lung maturity prior to the procedure due to the increased risk of preterm birth. Continuous fetal monitoring was performed throughout the surgery and was noted to be reassuring for the gestational age.

**Figure 2:**
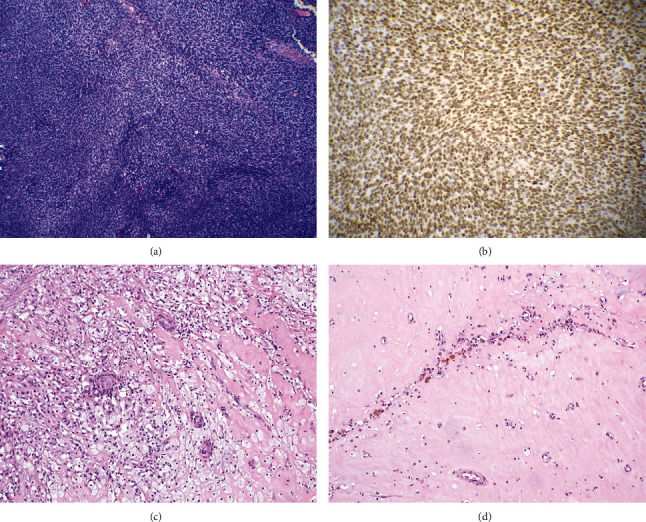
Histology of synovial sarcoma case. Diagnostic biopsy (a) demonstrated proliferation of spindle to epithelioid cells of monomorphic appearance with elongated nuclei and scant cytoplasm arranged in a whorled “herringbone” architecture (Hematoxylin and Eosin, 100x). Fli-1 immunohistochemistry (b) demonstrated strong, diffuse nuclear staining, confirming the diagnosis (200x). The posttreatment resection specimen demonstrated substantial treatment effected with the majority of residual tumor mass now comprised of areas of histiocytic infiltration (c) fibrosis, pigment deposition, and chronic inflammation (d) (Hematoxylin and Eosin, 20x).

Histopathology from the retroperitoneal biopsy demonstrated high-grade spindle cells with areas of herringbone pattern and small round blue cells (Figure 2). Frequent mitoses were noted (8/HPF). Immunohistochemistry (IHC) revealed tumor cells to be CD56+, bcl-2+, weakly TLE1+, partially CD34+, and focally S100+. Cells were negative for pankeratin, CAM 5.2, EMA, CD99, SOX10, SMA, desmin, synaptophysin, SALL4, CD10, CD31, CD117, WT1, and estrogen receptor. Fluorescence *in situ* hybridization (FISH) was positive for SYT (SS18) gene rearrangement. While the IHC profile was not specific, the morphologic features and positive SYT rearrangement result were consistent with a diagnosis of poorly differentiated synovial sarcoma, clinically staged IIIB (T3N0M0).

The case was discussed at a multidisciplinary sarcoma tumor board in consultation with Surgical Oncology, Medical Oncology, Radiology, Pathology, Teratology, and Maternal-Fetal Medicine with decision for neoadjuvant chemotherapy with doxorubicin and ifosfamide with modified dosing during the prepartum period as described in Mir et al [1]. The patient was admitted at 29w6d for cycle 1 of chemotherapy and at that time had a repeat baseline MRI which redemonstrated the now 15.5 × 10.2 × 10.6 cm mass encasing right renal pelvis and aorta, displacing left renal vessels posteriorly and IVC anterolateral (Figure 1). She received doxorubicin (50 mg/m^2^ day 1) and ifosfamide (2.5 g/m^2^/day, days 1-2) with standard mesna rescue without notable maternal side effects or fetal complications. She continued to follow closely for prenatal care and serial fetal growth ultrasounds. The timing for delivery was determined through multidisciplinary discussion with neonatology, maternal-fetal medicine, and oncology. Fetal antenatal testing was overall reassuring with normal fetal growth; however, the amniotic fluid index (AFI) was found to be borderline low (5.9 cm, with maximum vertical pocket (MVP) of 2.3 cm) and decreased from an AFI of 15.3 cm with an MVP of 5.7 cm prior to cycle 1 of neoadjuvant chemotherapy. Further, the patient had a prior history of two spontaneous preterm deliveries at approximately 36 wks. There was therefore a concern that delaying delivery for a second cycle of chemotherapy could result in a spontaneous delivery close to the administration of the chemotherapeutic agents which theoretically would increase the risk of neonatal adverse effects. Given the above considerations, the decision was made to proceed with a scheduled induction of labor (IOL) 3 wks after cycle 1 of neoadjuvant chemotherapy and to proceed with the remaining cycles postpartum. She was given a second course of corticosteroids for fetal lung maturity prior to her IOL, and she then underwent an uncomplicated IOL resulting in a vaginal delivery of a 1870 g (32nd percentile) male neonate with APGAR scores of 8 and 9 at 1 and 5 minutes, respectively. The patient had an uncomplicated postpartum course and was discharged home. The neonate's NICU course was complicated by mild respiratory distress syndrome requiring continuous positive airway pressure (CPAP) on admission which was weaned to room air by day of life (DOL) 7 as well as hyperbilirubinemia requiring 2 days of phototherapy. Otherwise, he had an uncomplicated NICU stay, was advanced to full PO feeds, monitored for temperature stability and adequate weight growth, and was discharged home on DOL 20 (corrected gestational age 36w0d).

The patient continued with neoadjuvant chemotherapy with cycle 2 beginning 1 week postpartum. She received doxorubicin (25 mg/m^2^/day, day 1-3) and ifosfamide (2.5 g/m^2^/day, day 1-4) with standard mesna rescue for the postpartum cycles. Restaging CT was performed following cycle 3 demonstrating marked decrease in the size of the retroperitoneal mass (Figure 1). She went on to complete three additional neoadjuvant chemotherapy cycles (6 total) without significant complication with restaging imaging again demonstrating substantial reduction in tumor size, then measuring 3.7 cm × 6.3 cm in greatest dimension (Figure 1).

Following completion of neoadjuvant chemotherapy, she received neoadjuvant external beam radiation therapy consisting of 5750 cGy in 25 fractions, which was completed without significant complication. She then went on to undergo radical resection of the retroperitoneal tumor including en bloc resection of infrarenal IVC and aorta and portion of omentum with 10 cm × 14 mm aortic reconstruction with tube graft and omental flap coverage of aortic prosthetic reconstruction. Her surgical recovery was unremarkable except for requirement of packed red blood cell transfusion twice for asymptomatic anemia with hemoglobin < 7 gm/dL on postoperative day numbers 3 and 7 with adequate response. She was discharged on postoperative day number 7 in good condition.

Pathology from the resection specimen demonstrated a 6 cm mass that on histologic evaluation exhibited marked treatment effect with only microscopic foci of residual synovial sarcoma in a background of necrosis, pigment deposition, histiocytic infiltrate, and inflammatory cells (Figure 2). The tumor focally invaded the soft tissue surrounding the proximal aortic resection margin with all other margins uninvolved by tumor.

Following resection of her sarcoma, the patient was followed with routine surveillance CT imaging including the chest, abdomen, and pelvis at 3-month intervals. Unfortunately, she developed an isolated pelvic recurrence 13-month postresection. As of this writing, she is undergoing salvage chemotherapy with neoadjuvant intent. Her 23-month-old son is healthy and has achieved appropriate developmental milestones.

## 2. Discussion

Synovial sarcomas (SS) are mesenchymal neoplasms that can occur throughout the body and account for 5-10% of all soft tissue sarcomas [2]. SS occur most common in the adolescent and young adult (AYA) age range with peak incidence in the third decade and hence can affect women of childbearing age. Presenting symptoms, particularly for retroperitoneal primary site locations, can often be vague and predate the diagnosis by years [2].

For nonmetastatic synovial sarcoma, surgery is the mainstay of therapy and only curative treatment. For higher risk tumors, traditionally defined by size ≥ 5 cm and high histologic grade, adjuvant or neoadjuvant therapy with radiation and/or systemic therapy is often considered for risk reduction of recurrence. Neoadjuvant treatment is increasingly utilized over adjuvant owing to the advantages of improved chance of conservative surgery as well as the ability to assess treatment response [3]. Regarding systemic therapy, synovial sarcoma is considered one of the most chemosensitive sarcoma subtypes and has among the strongest evidence for adjuvant/neoadjuvant chemotherapy use [4]. In the case described, even outside of the pregnancy considerations, there were strong indications for neoadjuvant chemotherapy given the high-risk features of the tumor and extensive visceral involvement complicating an initial surgical approach.

In review of the literature, there have been 13 previous cases on synovial sarcoma associated with pregnancy reported [5–18]. These patients, their treatment, and outcomes are summarized in Table 1. These cases occurred in various anatomical locations with two noted originating from soft tissue of the lower extremities, two from the oral cavity, three from the kidneys, one from the abdominal wall, and five from the chest. To our knowledge, ours is the first case reporting a synovial sarcoma originating in the retroperitoneum distinct from the renal structures during pregnancy. In only one of the previous reports was neoadjuvant chemotherapy administered antepartum.

For women diagnosed with malignancy during pregnancy, treatment decisions must balance optimal oncologic management with the interventional risks to both the mother and fetus. Regarding systemic chemotherapy, best available data suggests that the majority of agents evaluated do not appear to increase the risk of maternal or fetal complications when administered after the first trimester, nor appear to have long-term effects on children that were exposed *in utero* [19, 20]. Anthracycline-based chemotherapy combinations in particular have been fairly widely studied owing to their common use in the management of breast cancer and are considered relatively low risk for maternal and fetal complications [21]. Specific to sarcoma, prior literature regarding the use of neoadjuvant anthracycline plus ifosfamide chemotherapy is limited to one small series, suggesting this approach to be feasible and safe [1].

Systemic chemotherapy is often considered for treatment of malignancy in pregnancy, especially during the second and third trimesters after organogenesis is completed [22]. However, there are still risks of obstetric complications despite the completion of the majority of organogenesis including preterm delivery, fetal growth restriction, and intrauterine fetal demise [22]. While fetal growth restriction has been demonstrated in cases of malignancy in pregnancy, data are limited in suggesting whether the cause is related to toxicity of the systemic cytotoxic treatment or simply the pathophysiology of the disease itself. Van Calsteren et al. demonstrated higher rates of small for gestational age neonates in patients receiving cytotoxic treatment compared to those without (20.4 vs. 9.2%) [23]; however, Cardonick did not show increased rates of fetal growth restriction with cytotoxic treatment in their cohorts (7.6 vs. 7.1%) [24]. Data regarding ifosfamide use in pregnancy is particularly limited; however, Mir et al.'s review of ifosfamide use in pregnancy noted oligohydramnios in 5 of the 11 patients given this drug at various doses [1], suggesting this as a potential fetal toxicity and is notable in light of the borderline low AFI observed in our case that developed post-ifosfamide therapy. Among the cases of synovial sarcoma in pregnancy, two experienced fetal growth restriction [6, 9], and one was delivered for oligohydramnios with nonreassuring fetal monitoring [11]. These constellations of findings emphasize the importance of frequent fetal monitoring during treatment in order to detect potential obstetrical complications and modify delivery planning as indicated.

In summary, we report a unique case of a large, borderline resectable retroperitoneal synovial sarcoma presenting in a pregnant woman in the second trimester. This case highlights the need for multidisciplinary management in this high-risk scenario and argues for an aggressive treatment approach including administration of systemic chemotherapy in the antepartum setting to achieve optimal oncologic and maternal-fetal outcomes. This case, along with previous small series, suggests the potential for decreased amniotic fluid volumes associated with the use of ifosfamide, and therefore, frequent fetal monitoring in this scenario is recommended.

## Figures and Tables

**Table 1:** Synovial sarcoma cases in pregnancy.

Author/year	Patient presentation	Pathology	Oncologic treatment/outcome	Pregnancy treatment/outcome
Adamesteanu, 2015 [5]	19 yo presented to provider at 15 wks gestation for painful thigh lesion, clinically diagnosed as lipoma. Lesion grew throughout second and third trimesters. Referred for further evaluation postpartum.	Large biphasic synovial sarcoma invading surrounding fatty tissue, fascia, and tendons. IHC: vimentin+, B-cell lymphoma (bcl)+, epithelial membrane antigen (EMA) +; MIC occasionally +; Ki67 20%+. t (X; 18) (p11; q11) translocation unable to be tested.	Imaging evaluation for distant metastases was negative. Proceeded with left lower limb amputation with negative resection margins. Staged pT2bNx G3. Adjuvant chemotherapy with ifosfamide 5 g/m^2^ and doxorubicin 50 mg/m^2^ weekly x6 weeks. External radiotherapy 10 Gy/week for total dose of 50 Gy. Patient currently undergoing radiation, no evidence of local recurrence.	Full-term cesarean delivery. No treatment/evaluation during pregnancy.
Akhanoba, 2019 [6]	28 yo pregnant HIV+ female noted to have a mass on her left thigh. Ultrasound of thigh suspicious for sarcoma. MRI demonstrated large mass within left medial thigh (14 cm × 10 cm × 8 cm) replacing distal fibers of left adductor longus muscle belly, encasing femoral vessels, and infiltrating into left sartorius muscle.	Biopsy confirmed synovial sarcoma.	CT scan no evidence of distant metastases. Stage pT2bN0M0 G2. Initial surgery (hip disarticulation) at 30 wk gestation. 4 wks postoperative represented with sepsis and infected wound stump. Required three wound debridements and long-term IV antibiotics. Surveillance plan: clinical review and chest X-ray every 3 months x2 years then every 6 months x3 years, then annually x5 years.	Antenatal steroids given prior to initial surgery at 30 wk gestation. Presented at 34 wks with sepsis arising from infected leg stump. Fetal growth restriction suspected (EFW < 10% ile). Emergent cesarean and wound exploration and debridement at 34 wks. Delivered a female neonate weighing 1756 g.
Bettendorf, 2006 [7, 8]	32 yo G1P0 presented for evaluation of large left kidney mass after experiencing gross hematuria starting 3 weeks earlier. Renal ultrasound demonstrated abnormal mass concerning for neoplasm. MRI confirmed findings of a large tumor in the left lower renal pole. No lymphadenopathy, adrenal involvement, or distant metastases were noted.	Monomorphic cellular pattern with large cells and dark nucleoli. IHC vimentin+, bcl-2+, low growth ratio (MIB − 1 < 5%). CD 99-, estrogen receptor-, progesterone receptor-, AFP-, HCG-. RT-PCR confirmed t (X; 18) translocation with SYT-SSX2 fusion transcript confirming monophasic variant of synovial sarcoma.	Nephrectomy at time of cesarean delivery. Adjuvant chemotherapy with ifosfamide (5 g/m^2^) and doxorubicin (75 mg/m^2^) for two cycles followed by 54 Gy radiation to region. After radiation therapy, 2 additional cycles of ifosfamide/doxorubicin were given. Eight-month follow-up shows no recurrence.	34-week cesarean delivery with left nephrectomy after administration of antenatal corticosteroids.
Bunch, 2012 [9]	38 yo presented at 26 wk gestation with dyspnea, upper right back pain, and orthopnea. Chest X-ray and CT angiogram revealed large 12 cm mass in the right chest. CT-guided needle biopsy demonstrated high-grade neoplasm.	High-grade, poorly differentiated pulmonary synovial sarcoma	Right pneumonectomy and lymph node dissection performed at 28 wk gestation. Negative margins not obtained due to tumor burden. Patient presented 8 wks later with worsening dyspnea and new pulmonary embolism. Repeat CT revealed tumor growth and near complete occlusion of SVC. Radiation therapy administered. Unable to undergo chemotherapy due to liver dysfunction. Patient expired 2 weeks after readmission.	Antenatal steroids prior to 28 wk pneumonectomy. Fetal growth restriction noted at 30 wks with subsequent poor interval growth leading to repeat cesarean section at 32 wks. Newborn required pulmonary artery banding but was discharged home on day of life 50.
Esaka, 2008 [10]	G2P1 presented at 34 wks gestation with worsening dyspnea and left-sided cheat pain for 1 month. Chest X-ray revealed a left apical pneumothorax treated with pigtail chest tube. Repeat chest X-ray concerning for mass in this area. CT revealed left pneumothorax with significant complex mass in the left lung. Cardiothoracic surgery proceeded with video-assisted thoracoscopy.	Poorly differentiated synovial sarcoma within left pleural debris. IHC vimentin+, AW1/3+, CK7+, CK56+, K903+, S-100-, calretinin-.	Postpartum left pneumonectomy followed by adjuvant chemotherapy. Patient expired 13 months after resection.	Induction of labor after pathology findings confirmed neoplasm leading to a vacuum-assisted vaginal delivery of a 2125 g male neonate.
Harris, 2014 [11]	26 yo G2P1 at 21 wk gestation presented with dry cough and right sided pleuritic chest pain. CT revealed smooth, lobulated 6.5 cm mass in the right hilum. Bronchoscopy with multiple biopsies performed.	Malignant spindle cells infiltrating endobronchial tissue. IHC CD99+, bcl-2+, CD34-, AE1/3-, Cam5.2-, desmin-, smooth muscle actin-, S-100-, HMB45-. FISH revealed predominant population of polypoid cells with multiple copies of SYT probe. Findings consistent with monophasic spindle cell synovial sarcoma.	Primary surgical management deferred given pregnancy. Patient given 2 cycles of neoadjuvant chemotherapy: ifosfamide 2 g/m^2^ IV bolus over 4 h followed by 2 g/m^2^/day continuous IV for 6 days (total dose 14 g/m^2^/cycle) given with mesna. Cycles given at 26 and 29 wk gestation. CT scan postdelivery showed a 30% decrease in size of primary mass. Two additional neoadjuvant cycles with doxorubicin, ifosfamide, and mesna were given. Right total pneumonectomy and mediastinal lymphadenectomy performed 8 wk postpartum. Lymph nodes and margins negative. Patient declined adjuvant chemotherapy. 5 months later, recurrence occurred. Patient given palliative radiation. Patient expired 13 months after diagnosis.	Patient experienced decreased fetal movement at 31 wks and was diagnosed with oligohydramnios. She had a nonreassuring fetal heart tracing and was delivered via emergent cesarean delivery.
Kanade, 2013 [12]	21 yo G1 presented with slow growing mass in anterior abdominal wall. MRI revealed soft tissue mass attached to anterior abdominal wall without metastasis. FNA cytology revealed spindle cell tumor. Total excision of mass was then completed.	Crowded spindle cells in wavy, short fascicular pattern. IHC cytokeratin+, EMA+, Mic-2+, bcl-2+, calponin-. Pathological diagnosis of biphasic synovial sarcoma.	Patient declined local radiation therapy and chemotherapy. She presented 6 months later with local recurrence. She declined further treatment and was lost to follow-up.	No information available.
Khoja, 2013 [13]	23 yo at 28 wk gestation presenting with fever, cough, and dyspnea. Ultrasound revealed a large mass in left hemi-thorax. Initial biopsy inconclusive, repeat CT-guided biopsy revealed synovial sarcoma.	Information not available.	Advised for surgical resection and chemo-radiation therapy but further information not available.	32 wk cesarean delivery.
Nebhnani, 2014 [14]	25 yo at 8 wk gestation presented with abdominal distension, left flank pain, and hematuria x10 months. 10 cm flank mass palpated. CT/MRI consistent with renal cell carcinoma without evidence of metastases.	Malignant spindle cells arranged in sheets, fascicles, and whorls. IHC bcl2+, Mic2+, vimentin+. RT-PCR for fusion transcript SYT-SSX1 negative. Final diagnosis monophasic primary synovial sarcoma of the kidney.	Left radical nephrectomy referred to oncology for adjuvant chemotherapy.	Termination of pregnancy at 8 wks.
Orlandi, 2007 [15]	23 yo at 22 wk gestation presented with palpable lesion in the left cheek. She underwent surgical resection at 23 wks gestation with rupture of capsule intra-op.	Biphasic synovial sarcoma. IHC keratins+, EMA+, bcl-2+, CD99+. RT-PCR confirmed SYT-SSX1 fusion translocation.	Post-op MRI revealed possible residual disease and concern for high rate of recurrence given capsule rupture. Decision made to proceed with radiation therapy at 30 wk gestation with shielding of the abdomen/uterus. Patient received 48 Gy (24 of 25 fractions planned) prior to delivery with remainder after delivery. Eight months posttreatment without evidence of recurrent disease.	Planned cesarean delivery at 36 wks. 2800 g newborn male. Newborn healthy at 8 months of age.
Ortiz M, 2012 [16]	23 yo pregnant patient presenting with acute airway obstruction due to large hypopharynx lesion.	Synovial sarcoma.	Emergent tracheotomy and tumor debulking, partial pharyngectomy 6 weeks postpartum; no recurrence at 30-month post-op.	29-week preterm delivery.
Pathrose, 2017 [17]	25 yo G2P1 at 10 wk 4 d gestation was incidentally noted to have a left maternal renal mass on perinatal ultrasound. MRI revealed a 13 × 11 × 10 cm mass arising from the left kidney suggestive of renal cell carcinoma.	Tumor completely replaced the left kidney. Well circumscribed neoplasm of round to oval to spindle cells in sheets, fascicles, and perivascular arrangement. IHC TLE-1+, CD56+, CD99+, vimentin+, desmin-, myogenin-, EMA-, CK-, WT1-. Molecular analysis confirmed presence of SYT-SSX2 translocation.	Open left radical nephrectomy. Adjuvant chemotherapy: ifosfamide (1800 mg/m^2^) and doxorubicin (25 mg/m^2^) for 3 cycles. Disease free at 3-year follow-up.	Termination of pregnancy.
Sarkurai, 2006 [18]	33 yo female 5 months pregnant presented with acute onset back pain and was diagnosed with pleural effusion. Thoracentesis revealed frank blood. She deteriorated and required video-thoracoscopic evaluation with evacuation of 2000 cc of blood and multiple tumors attached to the parietal and visceral pleura including one that was bleeding and was resected for hemostasis and diagnosis.	Monophasic cells in plump, spindle-shaped appearance arranged in fascicles and herringbone pattern. Chromosomal translocation t (X; 18) (p11; q11) confirmed by FISH. Final diagnosis monophasic fibrous synovial sarcoma.	Patient refused further treatment after initial emergent surgery.	Abortion, not further specified.
